# Biomass and Abundance Biases in European Standard Gillnet Sampling

**DOI:** 10.1371/journal.pone.0122437

**Published:** 2015-03-20

**Authors:** Marek Šmejkal, Daniel Ricard, Marie Prchalová, Milan Říha, Milan Muška, Petr Blabolil, Martin Čech, Mojmír Vašek, Tomáš Jůza, Agustín Monteoliva Herreras, Lourdes Encina, Jiří Peterka, Jan Kubečka

**Affiliations:** 1 Biology Centre of the Academy of Sciences of the Czech Republic, v.v.i., Institute of Hydrobiology, České Budějovice, Czech Republic; 2 Faculty of Science, University of South Bohemia, České Budějovice, Czech Republic; 3 Ecohydros S.L., Polígono Industrial de Cros, Maliaño (Cantabria), Spain; 4 Department of Plant Biology and Ecology, Faculty of Biology, University of Seville, Seville, Spain; Bournemouth University, UNITED KINGDOM

## Abstract

The European Standard EN 14757 recommends gillnet mesh sizes that range from 5 to 55mm (knot-to-knot) for the standard monitoring of fish assemblages and suggests adding gillnets with larger mesh sizes if necessary. Our research showed that the recommended range of mesh sizes did not provide a representative picture of fish sizes for larger species that commonly occur in continental Europe. We developed a novel, large mesh gillnet which consists of mesh sizes 70, 90, 110 and 135mm (knot to knot, 10m panels) and assessed its added value for monitoring purposes. From selectivity curves obtained by sampling with single mesh size gillnets (11 mesh sizes 6 – 55mm) and large mesh gillnets, we identified the threshold length of bream (*Abramis brama*) above which this widespread large species was underestimated by European standard gillnet catches. We tested the European Standard gillnet by comparing its size composition with that obtained during concurrent pelagic trawling and purse seining in a cyprinid-dominated reservoir and found that the European Standard underestimated fish larger than 292mm by 26 times. The inclusion of large mesh gillnets in the sampling design removed this underestimation. We analysed the length-age relationship of bream in the Římov Reservoir, and concluded that catches of bream larger than 292mm and older than five years were seriously underrepresented in European Standard gillnet catches. The Římov Reservoir is a typical cyprinid-dominated water body where the biomass of bream > 292mm formed 70% of the pelagic trawl and purse seine catch. The species-specific relationships between the large mesh gillnet catch and European Standard catch suggested that the presence of carp (*Cyprinus carpio*), European catfish (*Silurus glanis*), tench (*Tinca tinca*) or bream warrants the use of both gillnet types. We suggest extending the gillnet series in the European Standard to avoid misinterpretation of fish community biomass estimates.

## Introduction

Developing a tool capable of producing an unbiased picture of lentic fish communities became a task of increasing urgency as biotic and abiotic characteristics of the environment started being evaluated by standardized methods across Europe [1,2]. In recent decades, gillnet sampling methodologies evolved from single mesh gillnets into multimesh gillnets and became one of the key tools for assessing fish biomass, abundance and species composition of lentic communities [2,3]. When compared to other methods for estimating fish abundance and biomass, gillnets have advantages such as relatively low sampling costs, ease of use and possible deployment in a variety of lentic water habitats [4]. The data obtained from standardized sampling procedures may be used for large scale comparisons across various water bodies and serves as the main tool for interpreting differences in fish communities [5–7]. During the development of lentic water metrics to support the Water Framework Directive [8], certain large fish species were included as indicators [2]. Because data obtained by European Standard gillnets (ESG hereafter) have limits in representative catchable size of fish [4], improvements to gillnet sampling methodology are required to avoid biased sampling results.

ESG data were recently used for analysing piscivore top down control of prey fish [5], and size spectra of lake fish assemblages [6,9]. ESG catch is used according to recent findings that fish smaller than 80mm are underrepresented [10–12] and are thus left out of subsequent size spectra analyses [6]. Although large fish are more likely to be caught in gillnets than their smaller conspecifics due to increasing swimming ability with body size [13,14], large fish are also reported to be underrepresented in standard gear catch [15–17].

Despite the usefulness of gillnet data in comparative studies, gillnets as a passive gear were repeatedly proven to be size- and species-selective [10,18,19]. Size- and species-selectivity may be based on different encounter, contact and retention probabilities [reviewed in 20]. The main nature of gillnet mechanical selectivity is based on the relationship between fish girth and mesh perimeter [21,22] or, alternatively, fish species length and mesh size [20,23,24]. In order to sample the wide range of fish sizes that compose a water body’s community structure, the multimesh gillnets of Norden type consisting of 12 different mesh sizes were developed [1]. To avoid biased length-frequency distribution of samples captured by multi-mesh gillnets, ESG mesh sizes follow a geometric series from 5mm to 55mm with a ratio of 1.25 [1,24] such that the selectivity of adjacent mesh sizes overlaps [25].

The efficiency of most sampling methods is affected by fish species and size. In various sampling methods, the lowest efficiencies have been recorded for extreme sizes of fish (i.e. very small and large individuals; [26]). This fact is also true for the ESG where a biased picture for young-of-the-year and one year old fish (so-called 0+ and 1+ fish) was detected. These small fish are underestimated by gillnet catches and need adjustment before the data can be correctly interpreted [10]. Jurvelius *et al*. briefly described biased gillnet catches of large fish in their comparison of four sampling methods (gillnetting, seining, trawling and hydroacoustics, [27]). The European Standard describes the catchable fish size as a range from 40–400mm total length [4]. An exact evaluation of the ESG catch of large fish has not yet been done and is missing from the scientific literature.

The goal of this study was to investigate how the ESG catch of large fish is biased in terms of fish biomass, abundance and fish species and size composition. In order to compare the ESG with another tool capable of estimating catch rates of large fish (biomass per unit of effort, BPUE), we simultaneously deployed ESG along with a novel type of multimesh gillnet consisting of four large mesh sizes (large mesh gillnet, LMG hereafter), expanding the geometric series of the European Standard. We also compared gillnet samples with catches obtained by pelagic trawling and purse seining that do capture the full spectrum of fish sizes. We anticipated that the largest fish of certain species would be underestimated by the ESG and that the LMG would improve their biomass estimates.

The aims of this study were: (I) to estimate the threshold fish size above which the ESG is ineffective for estimating fish community biomass and abundance using selectivity curves approach (II) to compare the fish community biomass spectrum obtained by ESG to that obtained by trawl, purse seine and LMG, (III) to identify species for which the LMG sampling is essential, and (IV) to analyse the sampling bias of ESG for large bream (*Abramis brama*), a common and widespread large fish species in Europe.

## Methods

### Fish sampling

Three types of gillnets were deployed during this study—single mesh gillnets (SMG hereafter), ESG and LMG. We used SMG and LMG data to describe mesh size selectivity and for fitting selectivity curves. ESG and LMG were deployed concurrently to directly compare their catches and to identify possible improvements of gillnet sampling by extending the mesh size range.

**Fig 1:**
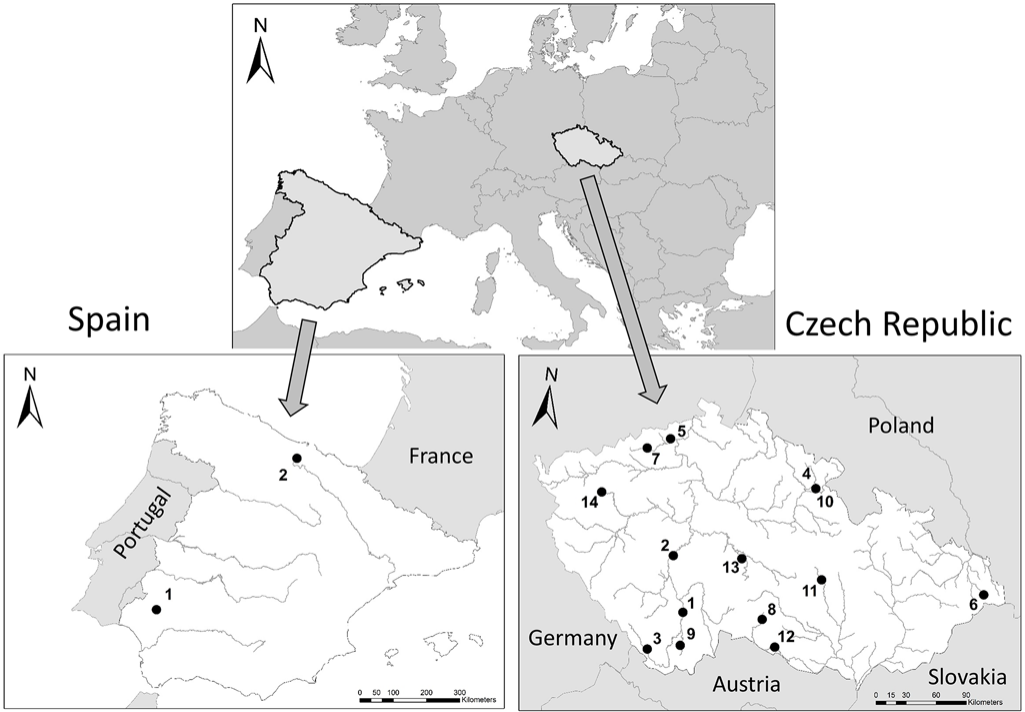
Map of the Czech Republic and Spain showing the locations of the water bodies sampled in this study. Reservoirs located in Spain: 1—Aracena, 2—Ebro. Reservoirs and lake located in the Czech Republic: 1—Hněvkovice, 2—Kamýk, 3—Lipno, 4—Malá Rozkoš 5—Milada, 6—Morávka, 7—Most, 8—Nová Říše, 9—Římov, 10—Rozkoš, 11—Vír, 12—Vranov, 13—Želivka, 14—Žlutice.

Sampling by SMG was conducted in the Římov Reservoir, Czech Republic, from 1999 to 2003. The mesh sizes used were 6.25, 8, 10, 12.5, 15.5, 19.5, 24, 29, 35, 43, 55, 60, 65 and 85mm (knot to knot; benthic and pelagic gillnet measured 1.5m height x 25m length and 4.5m height x 25m length, respectively, S1 Table). ESG following the European Standard Document [4] (benthic gillnet: 1.5m height x 30m length, 2.5m panels for each 12 mesh sizes; pelagic gillnet: 3m or 4.5m height x 30m length, 2.5m panels for each 12 mesh sizes) were used for sampling from 2009 to 2013 in 12 Czech and two Spanish reservoirs, and in two Czech post-mining lakes (Fig. 1, S2 Table). ESG mesh sizes follow a geometric series with a ratio of about 1.25 (5, 6.25, 8, 10, 12.5, 15.5, 19.5, 24, 29, 35, 43 and 55mm; [4]). LMG consisting of four mesh sizes extending the ESG geometric series (70, 90, 110 and 135mm; knot to knot; size 1.5m height x 40m length, 10m panels for each 4 mesh sizes) were deployed in the same habitats and localities along with the ESG. The density of large individuals is usually much lower than the density of their small conspecifics [15,28], so the length of LMG panels was made 4 times larger than ESG panels to increase the probability of capturing large individuals, and to improve the precision of abundance and biomass estimates. Both ESG and LMG were deployed in sets consisting of three gillnets joined by 30m rope. For LMG, mesh-specific catch was recorded in most years and water bodies.

Depth stratified sampling was conducted in all water bodies. Depth ranges were 0–3m, 3–6m, 6–9m, 9–12m, 12–18m and > 20m for benthic habitats; and 0–4.5m and 5–9.5m for pelagic habitats. Each water body was divided into several localities along its longitudinal axis in order to cover the gradient of fish distribution following nutrient concentration from tributary to dam [29]. Each locality was sampled to its maximum depth using the above stratification scheme. To cover both the sunset and sunrise peaks of fish activity, gillnets were deployed two hours before sunset and lifted two hours after sunrise [30].

The catch was sorted by species and standard length (SL) was measured for each individual fish. Catch data was expressed as biomass per unit of effort (BPUE, kilograms per 1000m^2^) by dividing the catch biomass by the area of deployed gillnets and multiplying by 1000. For each species, subsamples of at least 50 individuals from each reservoir and year were also weighed to the nearest gram to estimate length-weight relationships used in calculation of total catch biomass.

Active gear sampling was performed in the Římov Reservoir in 2010. Pelagic trawling and purse seining were used at night in the pelagic zone to obtain independent estimates of the length frequency distributions and biomass of the whole fish community. To obtain an unbiased sample of fish length-frequency distribution, density estimates of small fish (≤180mm) were obtained by purse seining and those of larger fish (>180mm) were obtained using the trawl [31]. This approach was chosen because of recent findings that the trawl used underestimates the fish density up to 180 mm SL [31]. The trawl opening width was 12–13.5m, and the opening height was 8m. The lengths of the main body and cod end were 38m and 11m, respectively. The mesh size in the main body was 80/40/20mm (half mesh, knot centre) from the opening toward the end and 10mm in the cod end. The purse seine net had a length of 120 m and height of 12 m. The exact position of each haul was recorded with a Garmin GPS device during the setting of the net, and the area sampled was calculated with OziExplorer software [32]. Trawl and purse seine catches were expressed as biomass per unit of effort (BPUE, kilograms per hectare).

### Selectivity curves

We calculated the intersection of selectivity curves of 55mm and 70mm mesh sizes for bream to identify the threshold standard fish length where sampling by the 70mm mesh size starts being more efficient than sampling with the 55mm mesh size. The intersection of the two curves is the fish length value *χ*_*threshold*_, which resulted in the same probability density for the Gaussian distributions for the 55mm and the 70mm mesh size. That is, we found the value *χ*_*threshold*_ such that:

$$f(x_{threshold},\mu_{55mm},\sigma_{55mm})=f(x_{threshold},\mu_{70mm},\sigma_{70mm})$$ (1)

where *f* is the Gaussian probability density function ($f(x,\mu ,\sigma )=\sqrt{\left(2\pi *\sigma^{2}\right)}*\text{exp}(-\frac{{\left(x-\mu \right)}^{2}}{2*\sigma^{2}})$, *μ*_55*mm*,_ *σ*_55*mm*_ are the mean and standard deviation of the 55mm mesh size selectivity curve and *μ*_70*mm*,_ *σ*_70*mm*_ are the mean and standard deviation of the 70mm mesh size selectivity curve.

### Biomass- and abundance-at-length

Biomass-at-length information was used to generate biomass spectra for ESG and LMG to determine the proportion of the fish community that was missed by ESG sampling. Length-specific BPUE (25mm length classes from 10 to 560mm) were computed for ESG and LMG and then used to compare the biomass spectrum captured by each type of gear, and to determine whether large fish were underrepresented and what improvements were achieved by the addition of LMG gear.

The length frequency distributions of fish caught by pelagic trawls and purse seines were compared to those obtained by ESG and LMG catches to evaluate whether the full spectrum of fish sizes was captured by using both ESG and LMG. We used a one-sided t-test to determine whether the mean fish length from the LMG was statistically smaller than the mean length of large fish captured by trawl (i.e. whether the LMG catch captures the whole size spectrum of large individuals). The trawl capture data used in the t-test consisted of fish individuals that were larger or equal to the smallest fish caught in the LMG.

To further examine whether the ESG captured the full spectrum of fish sizes, we calculated mesh-specific selectivity curves for bream sampled by SMG and LMG in the Římov Reservoir from 1999 to 2013. We chose bream as an example of a common and widespread species in Europe [33] because it has a wide spectrum of lengths over its long lifespan (see subsection Length-age analysis below) and is an ideal species to examine size selectivity of ESG. The catches obtained from three LMG mesh sizes (70, 90, 110mm) were used to determine whether or not some large fish were properly sampled by ESG.

To estimate whether biomass estimates derived from ESG samples were biased, and to quantify this bias, BPUE obtained from active gear and pelagic gillnet catches were compared for fish above and below the ESG standard length threshold (*SL≥χ*_*threshold*_ and *SL<χ*_*threshold*_). To circumvent the problem arising from the fact that trawl and gillnet BPUE have different units, BPUE ratios were used to provide a measure of ESG bias. We estimated the ESG bias for large fish by computing the difference in trawl to ESG BPUE ratios for small and large individuals using the following equation:

$$\frac{BPUE_{active,SL<x_{threshold}}}{BPUE_{ESG,SL<x_{threshold}}}=\beta_{ESG}\frac{BPUE_{active,SL\geq x_{threshold}}}{BPUE_{ESG,SL\geq x_{threshold}}}$$ (2)

where *β*_*ESG*_ quantified the ESG sampling bias magnitude for large fish. A positive value of *β*_*ESG*_ indicated that large fish were under-sampled by ESG. To determine whether the inclusion of LMG sampling reduced the estimated bias for large fish, a similar ratio was computed for trawl BPUE and total gillnet BPUE (ESG and LMG together) for fish above and below the ESG size threshold:

$$\frac{BPUE_{active,SL<x_{threshold}}}{BPUE_{ESG,SL<x_{threshold}}+BPUE_{LMG,SL<x_{threshold}}}=\beta_{ESG+LMG}\frac{BPUE_{active,SL\geq x_{threshold}}}{BPUE_{ESG,SL\geq x_{threshold}}+BPUE_{LMG,SL\geq x_{threshold}}}$$ (3)

where *β*_*ESG+LMG*_ is the estimated bias of the total gillnet catches.

### Species-specific LMG to ESG catch relationships

Species-specific benthic BPUE was computed for both ESG and LMG in all available reservoirs and years for the 17 species which had more than five BPUE records. The relationship between ESG and LMG biomasses (*ESG*_*s*_ and *LMG*_*s*_, respectively) was then estimated for every species *s* using the following linear model:

$$log(LMG_{s}+1)=\alpha_{s}+\beta_{s}log(ESG_{s}+1)$$ (4)

The model allows the identification of 1) species for which LMG catches are not significant (intercept *α*_*S*_ and slope *β*_*S*_ values near zero), 2) species for which LMG catches are positively correlated with ESG catches (intercept value near zero and positive slope value) and 3) species for which LMG catches are significant and are underrepresented in the ESG catches (positive intercept value). These species-specific estimates allowed the identification of the types of water bodies where LMG should be deployed along ESG to representatively sample all fish sizes.

### Length–age analysis

In order to evaluate the representation of large bream cohorts in terms of biomass and age classes between ESG and LMG, we examined the length-age structure of the Římov Reservoir bream population. 105 individuals caught during the 2012 sampling campaign were used for length-age analysis. Otolith reading was used for ageing fish. The relationship between *age* and *length* was estimated using a logarithmic curve of the form:

$$age=e^{\left(\alpha +\beta length\right)}$$ (5)

where *α* and *β* were estimated using a generalised linear model with Gamma error and a log link.

The estimated length-age relationship allowed us to identify the age of bream corresponding to a standard length of *χ*_*threshold*_ from equation (1). This standard length (*SL*) was then converted to total length (*TL*) using the following linear relationship:

TL=α+βSL (6)

where parameters *α* and *β* were estimated using 756 bream individuals for which both standard length and total length measurements were available. These individuals were captured during yearly sampling in the Římov Reservoir between 2000 and 2012. Standard lengths ranged from 31mm to 426mm and total lengths ranged from 39mm to 520mm.

All analyses were conducted using the R software version 3.1.2 [34].

## Results

From 1999 to 2003, a total of 1145 SMGs, with a combined sampled area of 60543.75m^2^, were deployed in the Římov Reservoir resulting in a catch of 14614 fish with a combined biomass of 1659.36kg. From 2009 to 2013, 1404 ESGs and 1221 LMGs were deployed in 16 water bodies with a combined sampled area of 93150 m^2^ and 108585 m^2^ respectively, resulting in a combined ESG catch of 84502 fish with biomass of 4082.25kg, and a combined LMG catch of 1396 fish with biomass of 2011.85kg.

The ESG catch contained a total of 36 species and 6 kinds of hybrids while the LMG catch consisted of 18 species and 2 hybrids (Table 1). However, the bulk of the LMG catch came from eight species: bream (55.2% in numbers and 31.7% in biomass), carp *Cyprinus carpio* (30.2% and 46.7%), European catfish *Silurus glanis* (2.1% and 5.8%), asp *Leuciscus aspius* (1.9% and 3.5%), tench *Tinca tinca* (2.9% and 3.2%), rudd *Scardinius erythrophthalmus* (2.6% and 2.0%), European whitefish *Coregonus lavaretus* (1.4% and 1.5%), and pikeperch *Sander lucioperca* (1.0% and 1.4%). Although ESG were deployed in the same reservoirs and localities as LMG, two species and a hybrid (bighead carp *Hypophthalmichthys nobilis*, Siberian sturgeon *Acipenser baerii* and white bream x rudd *Blicca bjoerkna x Scardinius erythrophthalmus* hybrid) were only detected by LMG (Table 1).

**Table 1:** Species captured by ESG and LMG in all water bodies sampled during the study.

Family	Common name	Scientific name	ESG	LMG
Acipenseridae	Siberian sturgeon	*Acipenser baerii*		X
Anguillidae	Eel	*Anguilla anguilla*	X	
Balitoridae	stone loach	*Barbatula barbatula*	X	
Centrarchidae	largemouth bass	*Micropterus salmoides*	X	
Centrarchidae	pumpkinseed	*Lepomis gibbosus*	X	
Cobitidae	spined loach	*Cobitis elongatoides*	X	
Cyprinidae	Andalusian barbel	*Luciobarbus sclaterii*	X	X
Cyprinidae	asp	*Leuciscus aspius*	X	X
Cyprinidae	belica	*Leucaspius delineates*	X	
Cyprinidae	bighead carp	*Hypophthalmichthys nobilis*		X
Cyprinidae	bleak	*Alburnus alburnus*	X	
Cyprinidae	bream	*Abramis brama*	X	X
Cyprinidae	carp	*Cyprinus carpio*	X	X
Cyprinidae	chub	*Squalius cephalus*	X	
Cyprinidae	dace	*Leuciscus leuciscus*	X	
Cyprinidae	Ebro barbel	*Luciobarbus graellsii*	X	X
Cyprinidae	Ebro nase	*Parachondrostoma miegii*	X	
Cyprinidae	Grass carp	*Ctenopharyngodon idella*	X	
Cyprinidae	gudgeon	*Gobio gobio*	X	
Cyprinidae	hybrid asp x ide	*Leuciscus aspius x Leuciscus idus*	X	
Cyprinidae	hybrid white bream x rudd	*Blicca bjoerkna x Scardinius erythrophthalmus*		X
Cyprinidae	hybrid white bream x vimba bream	*Blicca bjoerkna x Vimba vimba*	X	
Cyprinidae	hybrid white bream x bream	*Blicca bjoerkna x Abramis brama*	X	
Cyprinidae	hybrid roach x bream	*Rutilus rutilus x Abramis brama*	X	
Cyprinidae	hybrid white bream x roach	*Blicca bjoerkna x Rutilus rutilus*	X	
Cyprinidae	hybrid roach x rudd	*Rutilus rutilus x Scardinius erythrophthalmus*	X	X
Cyprinidae	Iberian gudgeon	*Gobio lozanoi*	X	
Cyprinidae	ide	*Leuciscus idus*	X	
Cyprinidae	Prussian carp	*Carassius gibelio*	X	X
Cyprinidae	roach	*Rutilus rutilus*	X	X
Cyprinidae	rudd	*Scardinius erythrophthalmus*	X	X
Cyprinidae	Spanish nase	*Pseudochondrostoma willkommii*	X	
Cyprinidae	stone moroko	*Pseudorasbora parva*	X	
Cyprinidae	tench	*Tinca*	X	X
Cyprinidae	vimba bream	*Vimba vimba*	X	
Cyprinidae	white bream	*Blicca bjoerkna*	X	X
Esocidae	pike	*Esox lucius*	X	X
Gobiidae	tubenose goby	*Proterorhinus marmoratus*	X	
Percidae	perch	*Perca fluviatilis*	X	X
Percidae	pikeperch	*Sander lucioperca*	X	X
Percidae	ruffe	*Gymnocephalus cernua*	X	
Salmonidae	brown trout	*Salmo trutta*	X	X
Salmonidae	European whitefish	*Coregonus lavaretus*	X	X
Salmonidae	grayling	*Thymallus thymallus*	X	
Salmonidae	rainbow trout	*Oncorhynchus mykiss*	X	
Siluridae	European catfish	*Silurus glanis*	X	X

### Selectivity curves

**Fig 2:**
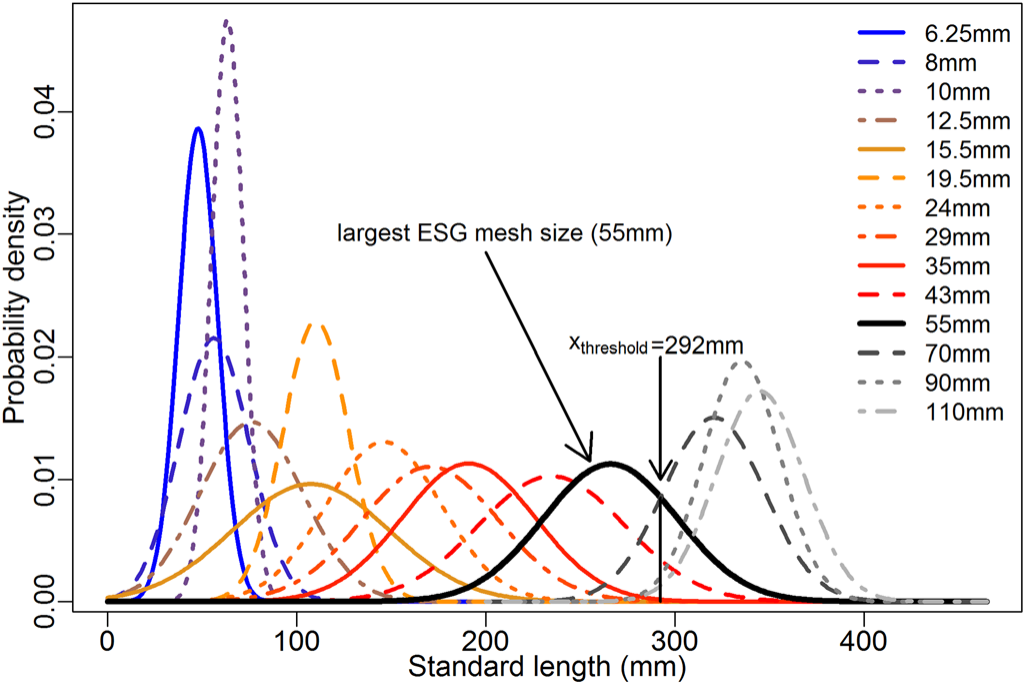
Mesh-specific length frequency distributions of bream captured in SMG in the Římov Reservoir and LMG in all sampled water bodies. The selectivity curve of the 55mm mesh size (the largest mesh size of the ESG) is highlighted and the fish standard length corresponding to the intersection between the 55 and 70mm curves (*χ*_*threshold*_) is shown.

From equation (1), we determined the intersection of the 55mm mesh size and 70mm mesh size Gaussian distribution curves to be 292mm for bream. The dataset used for length frequency analyses of bream included individuals ranging from 30 to 420mm in standard length. The majority of length classes were properly covered by mesh sizes used in ESG. Although there was a wide range of catchable lengths of ESG, ESG was not capable of enmeshing the larger bream also present in the community (Fig. 2). Thus, the ESG mesh size range did not representatively cover the whole size spectrum of bream.

### Biomass- and abundance-at-length

**Fig 3:**
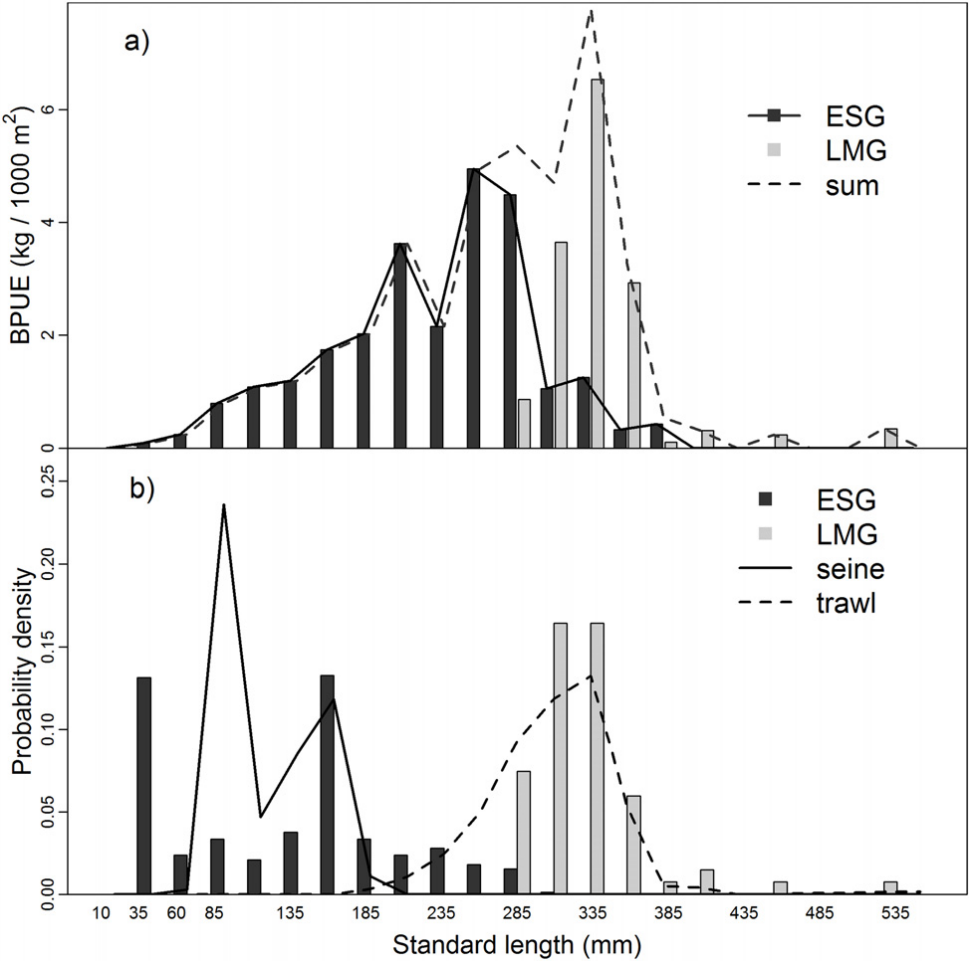
Biomass spectrum and length frequency distributions of all fish species captured in the Římov Reservoir in 2010. a) Biomass spectrum for ESG and LMG in the Římov Reservoir in 2010. b) Comparison of length frequency distributions of all fish species from trawl, purse seine, ESG and LMG sampling in the Římov Reservoir in 2010.

The biomass spectra (BPUE-at-length) for ESG and LMG obtained from the Římov Reservoir in 2010 (Fig. 3a) indicated that ESG sampling missed a significant proportion of the fish community biomass, especially for fish greater than 300mm standard length. While the ESG BPUE peaked at a standard length of 290mm and did not capture individuals larger than 390mm, the LMG BPUE peaked at a standard length of 330mm and extended towards larger sizes up to 530mm.

The LMG length-frequencies did not significantly differ from the large fish length-frequencies obtained by trawling (one-sided t-test, p = 0.94 in the 2010 sampling year). Both pelagic trawl and LMG showed peak frequencies for larger fish (300–350mm standard length, Fig. 3b). Fish above 300mm of standard length represented 37% of abundance and 70% of biomass of the trawled and purse seined fish community. However, this peak was not recorded in ESG catches indicating a serious underestimation of fish larger than 300mm of standard length during ESG sampling (Fig. 3b).

We estimated that the ESG bias for large fish, as determined by parameter *β*_ESG_ in equation (2), was a 26-fold underestimation. By adding the LMG samples to the sampling design (equation 3), the bias decreased substantially to a 1.36-fold underestimation of large fish. The value of both *β*_ESG_ and *β*_ESG+LMG_ represented the magnitude of the change in active gear to gillnet BPUE ratios for small and large fish. A value slightly above or below 1.0 can still identify an unbiased sampling strategy since there is a certain amount of measurement error in both gillnet and active gear catches.

### Species-specific LMG to ESG catch relationships

Based on species-specific regressions between LMG and ESG biomasses we identified species for which deployment of LMG significantly improved gillnet-based estimates. Small- and medium-sized species (bleak *Alburnus alburnus*, roach *Rutilus rutilus* and ruffe *Gymnocephalus cernua*) had intercept and slope values that were not statistically different from zero, indicating that they were never captured in LMG. Thus gillnet-based estimates of small- and medium-sized fish communities were unlikely to be improved by LMG sampling. Larger species (carp, European catfish, tench and bream) had significantly positive intercept (p<0.001, p<0.05, p<0.05 and p<0.1, respectively) and slope values (p<0.001, p<0.05, p<0.05 and p<0.01, respectively) indicating that these species were regularly recorded in LMG catches but underrepresented in ESG samples. A higher proportion of large species in fish communities led to a larger bias in the ESG estimates, and LMG sampling improved the biomass estimates of such communities. Rudd had a significantly positive slope (p<0.01) and near zero intercept value (p = 0.41, i.e. slope is not significantly different from zero), which suggested that its LMG catch was proportional to its ESG catch.

### Length-age analysis

**Fig 4:**
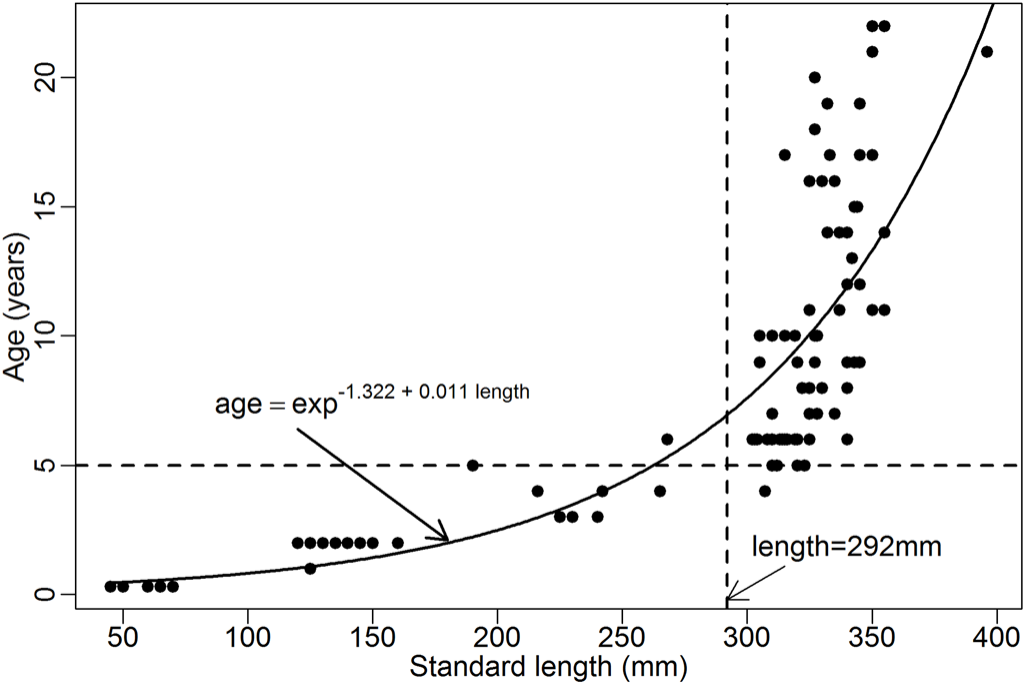
Bream length-age relationship in the Římov Reservoir, 2012. Vertical and horizontal lines indicate standard length of 292mm and age of five years, respectively, the threshold length and age above which bream is underrepresented in ESG catch.

The threshold standard length of bream (*χ*_*threshold*_ = 292*mm*) computed from equation (1) was equivalent to a total length of 366mm, (equation 6, *TL* = 4.4+1.24*SL*) which corresponded to 5 year old fish (Fig. 4). Bream larger than this threshold standard length of 292mm and 5 years of age and more were not representatively recorded by ESG sampling. These large individuals represented 10% of bream caught by SMG (231 out of 2290 individuals), and 35% of the bream catch weight (170 out of 486kgs). In comparison, large bream accounted for only 6% of individuals caught by ESG, but for 94% of individuals caught by LMG (38% and 96% of bream catch weight, respectively).

## Discussion

The current version of the European Standard document (2005) states that “the location of each gillnet in the lake is determined in such way that the total catch should constitute an unbiased sample of the catchable part of the fish assemblage in the lake. ‘*Catchable*’ fish means active fish species within a range of about 40mm to 400mm which are usually caught in gillnets.” We provided evidence of the fact that sampling by European Standard methods biases estimates of fish even smaller than 400mm of total length (367mm of total length in case of bream). Further, we showed that these fish constituted a considerable part of the fish assemblage in some water bodies. Because certain large fish species were recently included in the evaluation metrics of lentic waters within the Water Framework Directive [8], we recommend the addition of large-mesh gillnet sampling to avoid biased sampling results.

Obtaining a correct estimate of fish community length-frequency distribution requires representative fish samples. A major hurdle stems from the fact that all fishing gears provide biased results. The most pronounced biases in length-frequency distributions are for individuals of extreme sizes [26] and the largest individuals from a water body are often not detected by common sampling gear [16]. In the case of active fishing gear, large fish are able to escape from the moving gear because of their greater swimming capacity compared to smaller individuals [35,36]. Gillnet size-selectivity is strongly dependent on their design [37]. When comparing different gear for fish sampling, a number of studies identified an underestimation of large fish by ESG [15,17,27]. The latter study [17] suggests using 70mm mesh size gillnets in alpine lakes to increase the catchability of large fish and to improve estimates of size distribution and biomass of fish communities. We showed that simultaneous deployment of large mesh gillnet would improve the precision in length-frequency distribution estimates.

Holmgren & Appelberg [15] pointed out that additional sampling effort, beyond what is required for smaller fish, would be needed to obtain reliable biomass estimates for large fish. In the case of LMG design, we extended the length of large mesh panels from ESG’s 2.5m to 10m for several reasons. First, we expected that large fish would be less numerous than their smaller conspecifics due to the fact that natural mortality in fish is highest during early stages of their life history (so-called type III survivorship curve, [28]). Second, we assumed that gillnet saturation was more pronounced as the size of fish increase because of the disturbance that caught fish cause to their surroundings [38–40]. Third, we aimed to achieve a high precision in our estimates of large fish since large fish can compose a considerable part of the overall biomass in certain fish communities [27,41].

The composition of the fish community in a water body was shown to be mostly dependent on latitude, altitude, nutrient concentration and morphology [6,42,43]. The recent findings of Emmrich *et al*. [9] show that in general, average fish size declines from north to south. On a finer scale, we can identify several types of water bodies with distinct fish assemblages: deep vendace (*Coregonus albula*) lakes, shallow roach lakes and ruffe lakes [42]. A high proportion of large fish is found in alpine lakes [9,44], in bream-dominated lowland reservoirs of Central Europe and in northern shallow eutrophic lakes [45,46]. The lakes chosen for the development of ESG were based on sampling of predominantly small species [1,24,39]. Therefore, the largest mesh size from ESG geometric series is insufficient for sampling of large fish assemblages.

The data used in this study were collected predominantly in reservoirs, man-made water bodies with a number of anthropogenic pressures that are deteriorating aquatic communities [47]. Large fish are often the most prone to human influences and disappear from impacted ecosystems faster than small ones [48,49]. The large proportion of small fish observed while sampling reservoirs using gear that underestimates large fish may lead to the false assumption that large fish are only a minor part of the fish community. However, large fish are an integral part of ecosystem functioning [50] and may serve as an important indicator of human disturbances. Moreover, large trophy fish naturally attract the interest of the general public [16] and they have the potential to shape the rest of aquatic communities through predation [51] and very often represent the bulk of fish biomass [52]. For all the above reasons, the information about large fish must not be omitted in future standardised sampling.

33 European countries are currently using the European Standard or are bound to use it in near future [4]. Despite the fact that the standard is regularly debated by the European Committee for Standardization, there is a risk that such methodology could be implemented without proper evaluation of its potential shortcomings in water bodies where large fish are present. This study highlights that the European Standard underestimates the large fish community. Therefore, we suggest extending the European Standard mesh series with the additional larger mesh sizes used in this study. The large mesh gillnets should be used in water bodies where presence of larger fish species (e.g. bream, carp, European catfish, tench) is expected. The suggested modification to the sampling standard will ensure that the portion of the fish community that is composed of large fish is appropriately sampled and that biomass estimates for whole water bodies are representative of the entire fish community.

## Supporting Information

S1 Dataset Data file. Spreadsheet containing all the data required to reproduce the figures and analyses presented in the manuscript.
(XLSX)

S1 Table Single mesh gillnet catch and effort summary. Summary of gillnet effort (number of nets), sampled area and number of fish recorded in each SMG mesh size, Římov Reservoir, 1999–2003.
(DOCX)

S2 Table Multimesh gillnet catch and effort summary. Summary of the sampling in 2009–2013 in the different water bodies analysed in this study (CZ—Czech Republic, SP—Spain). For each water body, the year of sampling (Year), total gillnet effort (Nets), effort with ESG and LMG nets (ESG nets and LMG nets, respectively), the total number of fish (ESG and LMG fish) and biomass of fish recorded (ESG and LMG biomass in kg) are reported.
(DOCX)
