# Correction: Biomass and Abundance Biases in European Standard Gillnet Sampling

**DOI:** 10.1371/journal.pone.0128469

**Published:** 2015-05-11

**Authors:** Marek Šmejkal, Daniel Ricard, Marie Prchalová, Milan Říha, Milan Muška, Petr Blabolil, Martin Čech, Mojmír Vašek, Tomáš Jůza, Agustín Monteoliva Herreras, Lourdes Encina, Jiří Peterka, Jan Kubečka

The following information is missing from the Funding section: This study received institutional support from the Czech Academy of Sciences (RVO:60077344, http://www.avcr.cz/) and the CEKOPOT project (CZ.1.07/2.3.00/20.0204), co-financed by the European Social Fund and the state budget of the Czech Republic. Additionally, this study was supported by the Grant Agency of the Czech Republic (Project GPP505/12/P647). Daniel Ricard was supported by project CZ.1.07/2.3.00/30.0032 (Promotion of postdoctoral positions in the Biology Centre of the Czech Academy of Sciences), co-financed by the European Social Fund and the state budget of the Czech Republic.

The complete, correct funding statement should read: This study received institutional support from the Czech Academy of Sciences (RVO:60077344, http://www.avcr.cz/) and the CEKOPOT project (CZ.1.07/2.3.00/20.0204), co-financed by the European Social Fund and the state budget of the Czech Republic. Additionally, this study was supported by the Grant Agency of the Czech Republic (Project GPP505/12/P647). Daniel Ricard was supported by project CZ.1.07/2.3.00/30.0032 (Promotion of postdoctoral positions in the Biology Centre of the Czech Academy of Sciences), co-financed by the European Social Fund and the state budget of the Czech Republic. Ecohydros S.L. provided support in the form of salaries for authors [AMH], but did not have any additional role in the study design, data collection and analysis, decision to publish, or preparation of the manuscript. The specific roles of these authors are articulated in the ‘author contributions’ section.
