# Supplementary material for: Biomass and Abundance Biases in European Standard Gillnet Sampling
Source: PLoS One. 2015 Mar 20;10(3):e0122437. doi: 10.1371/journal.pone.0122437 (PMC4368622; doi:10.1371/journal.pone.0122437)
Supplement: S1 Table — Summary of gillnet effort (number of nets), sampled area and number of fish recorded in each SMG mesh size, Římov Reservoir, 1999–2003. (DOCX) [file pone.0122437.s002.docx]

**S1_Table: Single mesh gillnet catch and effort summary.** Summary of gillnet effort (number of nets), sampled area and number of fish recorded in each SMG mesh size, Římov Reservoir, 1999-2003.

| Mesh size (mm) | Number of nets | Sampled area (m^2^) | Number of fish | Catch biomass (kg) |
| --- | --- | --- | --- | --- |
| 6.25 | 91 | 2625.00 | 472 | 3.12 |
| 8 | 74 | 2662.50 | 1527 | 10.05 |
| 10 | 94 | 3881.25 | 1564 | 18.10 |
| 12.5 | 78 | 3506.25 | 1397 | 28.98 |
| 15.5 | 96 | 5231.25 | 2080 | 91.27 |
| 19.5 | 85 | 5212.50 | 1869 | 107.86 |
| 24 | 94 | 5625.00 | 1535 | 173.39 |
| 29 | 85 | 5212.50 | 1434 | 243.40 |
| 35 | 96 | 5587.50 | 1289 | 297.49 |
| 43 | 85 | 5212.50 | 771 | 279.62 |
| 55 | 95 | 5625.00 | 449 | 211.36 |
| 60 | 5 | 450.00 | 42 | 23.98 |
| 65 | 78 | 4687.50 | 146 | 116.74 |
| 85 | 87 | 5025.00 | 39 | 53.99 |
| Total: | 1145 | 60543.75 | 14614 | 1659.36 |
