# Supplementary material for: Biomass and Abundance Biases in European Standard Gillnet Sampling
Source: PLoS One. 2015 Mar 20;10(3):e0122437. doi: 10.1371/journal.pone.0122437 (PMC4368622; doi:10.1371/journal.pone.0122437)
Supplement: S2 Table — Summary of the sampling in 2009–2013 in the different water bodies analysed in this study (CZ—Czech Republic, SP—Spain). For each water body, the year of sampling (Year), total gillnet effort (Nets), effort with ESG and LMG nets (ESG nets and LMG nets, respectively), the total number of fish (ESG and LMG fish) and biomass of fish recorded (ESG and LMG biomass in kg) are reported. (DOCX) [file pone.0122437.s003.docx]

**S2_Table: Multimesh gillnet catch and effort summary.** Summary of the sampling in 2009 – 2013 in the different water bodies analysed in this study (CZ – Czech Republic, SP – Spain). For each water body, the year of sampling (Year), total gillnet effort (Nets), effort with ESG and LMG nets (ESG nets and LMG nets, respectively), the total number of fish (ESG and LMG fish) and biomass of fish recorded (ESG and LMG biomass in kg) are reported.

| Water body | Year | Nets | ESG nets | LMG nets | Benthic gillnets | | | | | | Pelagic gillnets | | | | | |
| --- | --- | --- | --- | --- | --- | --- | --- | --- | --- | --- | --- | --- | --- | --- | --- | --- |
|  |  |  |  |  | ESG nets | ESG fish | ESG biomass | LMG nets | LMG fish | LMG biomass | ESG nets | ESG fish | ESG biomass | LMG nets | LMG fish | LMG biomass |
| Aracena (SP) | 2009 | 60 | 30 | 30 | 30 | 2039 | 244.18 | 30 | 22 | 26.05 |  |  |  |  |  |  |
| Ebro (SP) | 2009 | 42 | 21 | 21 | 21 | 2041 | 94.08 | 21 | 63 | 187.13 |  |  |  |  |  |  |
| Hněvkovice (CZ) | 2013 | 114 | 57 | 57 | 36 | 1951 | 132.68 | 36 | 60 | 75.48 | 21 | 1615 | 171.45 | 21 | 54 | 66.13 |
| Kamýk (CZ) | 2012 | 66 | 33 | 33 | 21 | 96 | 16.42 | 21 | 34 | 76.36 | 12 | 91 | 15.04 | 12 | 52 | 103.93 |
| Lipno (CZ) | 2009 | 81 | 45 | 36 | 45 | 1892 | 115.92 | 36 | 23 | 34.76 |  |  |  |  |  |  |
|  | 2010 | 129 | 69 | 60 | 48 | 1088 | 97.46 | 39 | 37 | 56.79 | 21 | 654 | 65.27 | 21 | 20 | 32.62 |
|  | 2012 | 120 | 60 | 60 | 39 | 2495 | 114.40 | 39 | 21 | 34.62 | 21 | 1287 | 81.74 | 21 | 27 | 62.85 |
| Malá Rozkoš (CZ) | 2013 | 36 | 18 | 18 | 12 | 4110 | 75.38 | 12 | 81 | 128.33 | 6 | 4061 | 58.08 | 6 | 61 | 85.98 |
| Milada (CZ) | 2009 | 90 | 45 | 45 | 45 | 4848 | 107.64 | 45 | 5 | 11.11 |  |  |  |  |  |  |
|  | 2010 | 120 | 60 | 60 | 45 | 868 | 62.24 | 45 | 9 | 14.98 | 15 | 116 | 17.47 | 15 | 5 | 11.00 |
|  | 2012 | 120 | 60 | 60 | 45 | 1815 | 109.16 | 45 | 41 | 61.61 | 15 | 336 | 14.56 | 15 | 1 | 1.29 |
| Morávka (CZ) | 2012 | 72 | 36 | 36 | 27 | 2667 | 161.07 | 27 | 5 | 19.92 | 9 | 457 | 47.89 | 9 | 1 | 0.28 |
| Most (CZ) | 2011 | 126 | 63 | 63 | 45 | 9977 | 242.69 | 45 | 2 | 2.24 | 18 | 909 | 47.90 | 18 |  |  |
|  | 2012 | 126 | 69 | 57 | 45 | 2162 | 162.50 | 45 | 17 | 24.55 | 24 | 371 | 39.44 | 12 |  |  |
| Nová Ríše (CZ) | 2013 | 66 | 33 | 33 | 21 | 545 | 35.90 | 21 | 4 | 7.00 | 12 | 349 | 23.50 | 12 | 1 | 4.67 |
| Římov (CZ) | 2010 | 150 | 75 | 75 | 51 | 1112 | 75.61 | 51 | 52 | 47.36 | 24 | 358 | 29.60 | 24 | 70 | 82.41 |
|  | 2011 | 150 | 75 | 75 | 51 | 943 | 79.52 | 51 | 32 | 32.32 | 24 | 650 | 53.22 | 24 | 21 | 21.77 |
|  | 2012 | 144 | 72 | 72 | 51 | 1247 | 55.87 | 51 | 33 | 26.31 | 21 | 1170 | 69.16 | 21 | 92 | 108.65 |
|  | 2013 | 162 | 84 | 78 | 54 | 1346 | 87.64 | 54 | 44 | 41.85 | 30 | 784 | 69.10 | 24 | 76 | 70.42 |
| Rozkoš (CZ) | 2013 | 78 | 39 | 39 | 24 | 2481 | 102.10 | 24 | 104 | 133.07 | 15 | 599 | 35.92 | 15 | 24 | 25.79 |
| Vír (CZ) | 2010 | 69 | 57 | 12 | 39 | 3526 | 106.36 |  |  |  | 18 | 5179 | 67.60 | 12 | 14 | 34.28 |
|  | 2011 | 63 | 54 | 9 | 39 | 1445 | 90.81 |  |  |  | 15 | 756 | 52.76 | 9 | 14 | 16.27 |
| Vranov (CZ) | 2011 | 81 | 69 | 12 | 45 | 1767 | 130.30 |  |  |  | 24 | 2001 | 115.60 | 12 | 106 | 91.34 |
| Želivka (CZ) | 2010 | 150 | 75 | 75 | 51 | 4810 | 114.36 | 51 | 26 | 38.21 | 24 | 3419 | 67.55 | 24 | 11 | 25.6 |
| Žlutice (CZ) | 2011 | 102 | 51 | 51 | 36 | 653 | 110.97 | 36 | 7 | 20.76 | 15 | 149 | 54.70 | 15 | 8 | 33.85 |
|  | 2012 | 108 | 54 | 54 | 39 | 1135 | 114.51 | 39 | 12 | 23.51 | 15 | 132 | 44.94 | 15 | 4 | 8.38 |
| Total: |  | 2625 | 1404 | 1221 | 1005 | 59059 | 2839.77 | 864 | 734 | 1124.32 | 399 | 25443 | 1242.49 | 357 | 662 | 887.51 |
